# Psychological stress is related to a decrease of serum anti-müllerian hormone level in infertile women

**DOI:** 10.1186/s12958-017-0271-4

**Published:** 2017-07-11

**Authors:** Yue-zhi Dong, Fei-jing Zhou, Ying-pu Sun

**Affiliations:** Reproductive Medicine Center, The First Affiliated Hospital of Zhengzhou University, 1 Jianshedong Road, Erqi District, Zhengzhou, Henan Province 450052 China

**Keywords:** Stress, Anti-müllerian hormone, Ovarian reserve, Infertility

## Abstract

**Background:**

Stress exposure has been proved to be linked to reproductive failure. The reproductive potential of women depends on the ovarian reserve. Anti-müllerian hormone (AMH) has been proved a reliable clinical marker of ovarian reserve. However, the correlation between psychological stress and AMH level is not clear.

**Methods:**

A cross-sectional study including 576 women was conducted. AMH concentration was tested to reflect the ovarian reserve. Salivary alpha-amylase (SAA) level was measured to assess the stress of patients objectively.

**Results:**

The SAA level was significantly, and negatively correlated with AMH levels in infertile women (*r* = −0.315, *P* = 0.000; adjusted for age, *r* = −0.336, *P* = 0.000).

**Conclusion:**

Higher psychological stress was related to a decreased AMH level in infertile women and psychological stress may affect ovarian reserve.

## Background

With the change of society, economy, culture, living environment and working rhythm, females tend to be under increasing pressure. In fact, stress exposure has been proved to be linked to reproductive failure. Lynch et al. reported that preconception stress was associated with a longer time-to-pregnancy and an increased risk of infertility [1]. Infertility itself is a rather stressful event for patients, especially for women [2, 3]. Infertility-induced stress exerts a negative effect on the fertility treatment outcome directly or indirectly [4–6]. Impaired reproductive outcomes may be triggered by stress-inducing events and may be more prevalent in women susceptible to a physiological stress over-response [7].

The reproductive potential of women depends on the ovarian reserve Good ovarian reserve indicates more follicles and a high success rate in assisted reproductive technology (ART). Anti-müllerian hormone (AMH) is a glycoprotein secreted by the granulosa cells of small growing follicles and has been proved a reliable clinical marker of ovarian reserve [8, 9]. Compared with age, follicle-stimulating hormone (FSH) levels on day 3 of the menstrual cycle, estradiol (E2) levels or inhibin levels, AMH is a better marker in predicting ovarian response to controlled ovarian stimulation (COS) prior to in vitro fertilization (IVF) and intracytoplasmic sperm injection (ICSI) [10]. Some studies also showed that AMH could be a predictor of the supernumerary good-quality blastocysts for cryopreservation, and even the success rates of IVF [11, 12]. These studies suggested that AMH is an ideal marker for reproductive potential and outcomes. However, the correlation between psychological stress and AMH level is not clear.

Salivary alpha-amylase (SAA) is produced by the serous acinus cells of the parotid, submandibular and sublingual glands, which accounts for 40% to 50% of the total salivary gland-produced protein [13]. The production of this enzyme is controlled by the sympathetic and parasympathetic nervous systems. Chronic stress could activate the sympathetic adrenomedullary (SAM) pathway, and promote norepinephrine secretion into the bloodstream, resulting in an increase in SAA [14]. SAA has been proven to be a sensitive bio-marker of psychological stress, which could been detected in saliva in population-based studies [14, 15].

In this study, we will examine AMH levels in infertile women, assess their stress through SAA and analyze whether psychological stress is related to serum AMH level in infertile women.

## Methods

### Subjects

All participants were recruited from the assisted reproduction center of the First affiliated hospital of Zhengzhou University, China. Inclusion criteria included: (1) aged 23-45 years; (2) body mass index (BMI) < 24 kg/m^2^; (3) self-reported menstrual cycle length of 21–42 days; (4) receiving the first ART treatment; (5) not taking contraceptive, hypertensive and hormonal drugs in the past 3 months. (6) no history of thyroid condition or mental illness; (7) no history of uterine or ovarian surgery. From March 2016 to October 2016, 611 women met the inclusion criteria. All subjects were informed about the objective of this study and the informed consent was signed. Twenty-two women refused it due to lack of interest and 13 women were excluded because of substandard saliva samples. Finally, 576 infertile women joined this study. The saliva and blood samples were collected. The study was approved by the institutional ethics committee of Zhengzhou University, China.

### Anti-Müllerian hormone level

Generally, serum hormones of subjects were detected at day 2–4 of the menstrual cycle as basal hormone levels. To assess the basal AMH, two milliliters of blood samples were collected aseptically from the subjects in day 2–4 of the menstrual cycle. After centrifugation, serum was analyzed by an electrochemical luminescence analyzer (Roche, Cobas e601, Canada) for detection of AMH level (ng/ml). The theoretical sensitivity of the method is 0.006 ng/ml. Three women had undetectable AMH levels which were analyzed as 0.00 ng/ml in this study.

### Psychological stress

To assess stress objectively, SAA level was measured in this study. Saliva was collected using a salivate collection device before entering into their first treatment cycles. SAA and AMH samples were collected on the same day from each woman. Women were told to collect the sample between 8:00 and 11:00 am before eating, drinking, smoking or brushing their teeth for at least 2 h [16]. Samples were stored at −20 °C until tested. Salivary alpha-amylase level (μmol/L) was measured using an enzyme-linked immunosorbent assay (ELISA) kit (Labsystems Multiskan MS 352, Finland). The theoretical sensitivity of the method is 1.20 μmol/L. All subjects had detectable SAA levels in this study.

### Statistical analysis

Data management and analysis were performed using SPSS 19.0 (SPSS Inc., Chicago, IL, USA). Female fecundity decreases with the increasing chronological age. Based on the advanced reproductive age, female subjects were divided into two groups: <35 years and >35 years of age. *T*-test was used to analyze the statistical difference in SAA and basal AMH levels between the two groups. Correlation scatter diagram and Pearson correlation were used to analyze the relationship between the SAA and AMH levels. *P* < 0.05 was considered statistically significant.

## Results

### The distribution of SAA and AMH levels with age

**Fig. 1:**
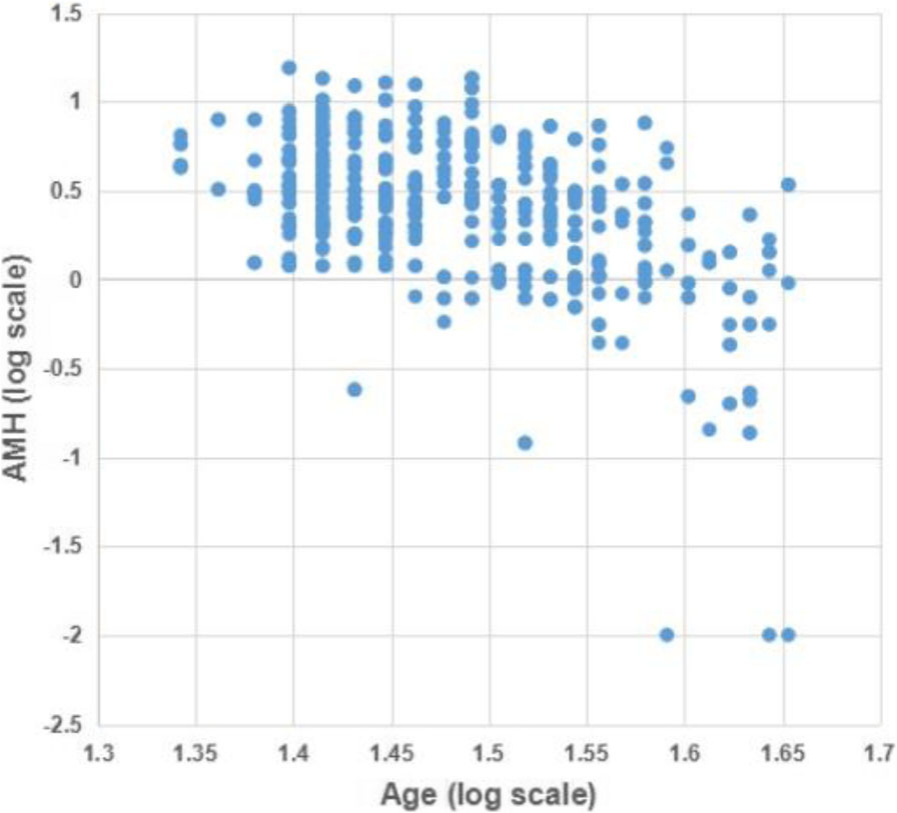
The distribution of AMH levels with age

In total, 576 infertile women participated in this study. Among them, 258 women were diagnosed as primary infertility while 318 as secondary infertility. The mean age of subjects was 32.3. 23.6% were over 35 years old. The duration of infertility was 4.8 years on average, ranging from 1 year to 13 years. Figure 1 showed that AMH level decreased with the increasing chronological age (*r* = −0.391, *P* = 0.000). Based on the advanced reproductive age, female subjects were divided into two groups: <35 years and >35 years of age. AMH level decreased significantly in women over 35 years (4.02 ± 2.69 VS 1.77 ± 1.61, *t* = 9.228, *P* = 0.000). However, no significant differences were found between the two groups in SAA (139.06 ± 42.63 VS 140.06 ± 40.62, *t* = −0.243, *P* = 0.808).

### Correlation between psychological stress and the Anti-Müllerian hormone levels

**Fig. 2:**
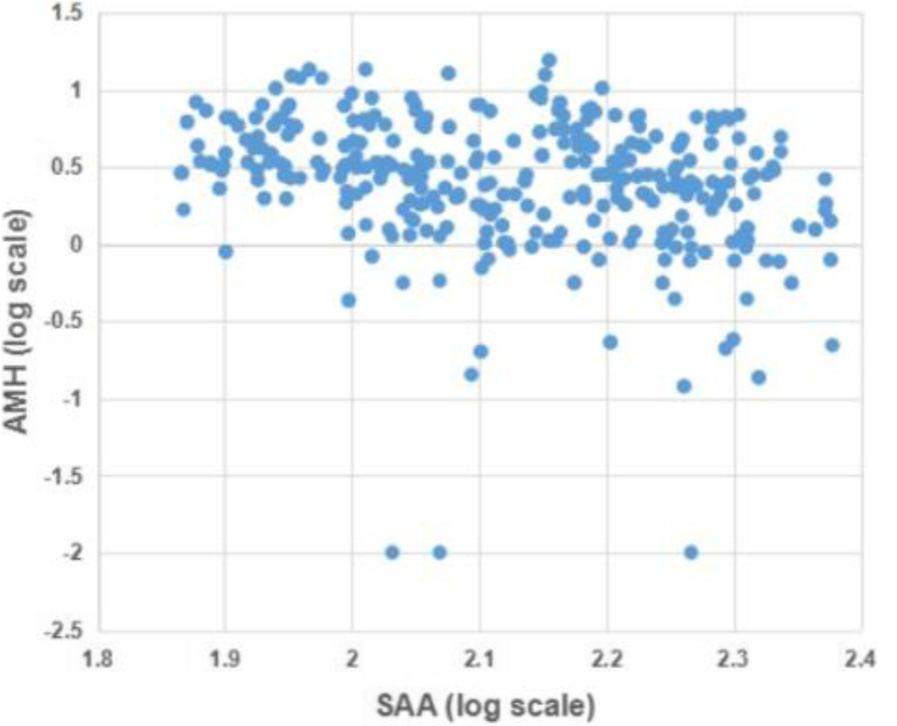
Correlation scatter diagram of the SAA and AMH levels. “*r*” means correlation coefficient; *P*<0.05 was considered statistically significant. *r* = -0.315, *P* = 0.000; adjusted for age, *r* = -0.336, *P* = 0.000

We further analyzed the correlation between psychological stress and the AMH levels. Figure 2 showed the correlation scatter diagram of the SAA and AMH levels in infertile women. Pearson correlation analysis showed there was a significant and negative correlation between the SAA and AMH levels in women (*r* = −0.315, *P* = 0.000; adjusted for age, *r* = −0.336, *P* = 0.000).

## Discussion

Studies showed that stress exposure was related to reproductive failure [1]. In this study, we found that there was a significant correlation between psychological stress and decreased AMH levels for infertile women.

AMH is produced by the growing follicles, and the levels of serum AMH are positively related to ovarian follicle numbers [17]. Researchers demonstrated that AMH could inhibit the depletion of primordial follicle and preserve the ovarian reserve [18]. Good ovarian reserve is the basis of fertility in women. For women, the primordial follicles are partially recruited into the cycle continuously, The reproductive potential for women are close to the quantity and quality of the primordial follicles remaining in the ovaires. The recent meta-analysis by Broer et al. also suggested that AMH can be used to predict excessive responses during controlled ovarian hyperstimulation (COH) [19]. Van Rooij et al. reported that AMH levels correlate with the age-related decline in reproductive capacity [20]. In our study, we found that the AMH levels decreased significantly in women over 35 years. So serum AMH levels were a valid indicator for reproductive potential.

Lynch et al. reported that preconception stress was associated with a longer time-to-pregnancy and an increased risk of infertility [1]. Some studies showed that chronic stress might affect the female reproductive system through hypothalamus-pituitary-adrenal (HPA) axis and sympathetic adrenomedullary (SAM) pathway [21–24]. When a stimulus is perceived as stressful, the HPA axis and SAM pathway may be activated [1]. As a result, growing follicles are lost due to the oxidative damage of ovarian follicle cells, which leads to a decrease of AMH level. In this study, we examined the AMH level and salivary alpha-amylase level in infertile women to analyze whether psychological stress influences the AMH level.

The present study was limited in that SAA was the only measure of stress. An SAA cut-off to establish high stress has yet to established. Further examination of stress levels in women, by life event analysis and/or surveys, in relation to serum AMH levels are the next step in exploring this topic.

## Conclusion

In summary, the results of this study suggest that higher psychological stress, measured as SAA levels, was related to a lower serum AMH level in infertile women. The effects of psychological stress on ovarian reserve needs to be studied further. Regardless, attention should be paid to relieve stress to promote reproductive health of women.

## Abbreviation

AMH: Anti-müllerian hormone
ART: Assistant reproduction technology
BMI: Body mass index
COH: Controlled ovarian hyperstimulation
COS: Controlled ovarian stimulation
E_2_: Estradiol
FSH: Follicle-stimulating hormone
HPA: Hypothalamus-pituitary-adrenal
ICSI: Intracytoplasmic sperm injection
IVF: In vitro fertilization
SAA: Salivary alpha-amylase
SAM: Sympathetic adrenomedullary
